# Data on length-weight and length-length relationships, mean condition factor, and gonadosomatic index of *Rutilus rutilus* and *Perca fluviatilis* from the Ob River basin, Western Siberia

**DOI:** 10.1016/j.dib.2022.108067

**Published:** 2022-03-18

**Authors:** Leonid A. Shuman, Aleksandr G. Selyukov, Innokenty S. Nekrasov, Andrey V. Elifanov, Victoria V. Yurchenko

**Affiliations:** a AquaBioSafe Laboratory, University of Tyumen, Volodarskogo St. 6, Tyumen 625003, Russia; b Institute of Biology, University of Tyumen, Russia

**Keywords:** LWR, LLR, GSI, Linear regression, Fish, Roach, Perch, CF, condition factor, EW, eviscerated weight, GSI, gonadosomatic index, GW, gonads weight, LLR, length-length relationship, LWR, length-weight relationship, SL, standard length, TL, total length, TW, total weight

## Abstract

Raw data were obtained from 250 specimens of roach *Rutilus rutilus* and 274 specimens of perch *Perca fluviatilis* at 15 sampling sites located from North to South Taiga. Fish sampling was performed using gill-nets with 22 and 28 mm mesh. Total and eviscerated weights, total and standard lengths, fish sex, gonad weight and maturity stage were recorded. Linear regression analysis of log-transformed total weight and total length values was performed. Regression slope and intercept were used to obtain length-weight relationships and mean condition factor values. Length-length relationships were calculated by the linear regression between the total and standard lengths. Gonadosomatic index was determined using the gonad weight and the total weight of fish. The data are useful for establishing biomass, fish growth patterns, relative condition of individuals within a sample or across populations.

## Specifications Table

**Table:** 

Subject	Animal Science and Zoology
Specific subject area	Ichthyology, fisheries
Type of data	Table Graph
How data were acquired	A measuring board and an Adam HCB 1002 digital balance (Adam Equipment, UK) were used to acquire raw data. Analysis was performed using Microsoft Office Excel software.
Data format	Raw Analyzed
Parameters for data collection	Roach *Rutilus rutilus* and European perch *Perca fluviatilis*, abundant fish species inhabiting lakes and rivers [1], were chosen for data collection. The latitudinal distribution of sampling sites within the Ob River basin was considered for data collection.
Description of data collection	Total and eviscerated weights, total and standard lengths, sex, gonad maturity stage and weight were recorded. Length-weight relationships were calculated by the linear regression of log_10_-transformed total weight on total length. Length-length relationships were obtained by the linear regression of total length on standard length. The mean condition factor was calculated using parameters of length-weight relationships. Gonad weight and the total weight of fish were used to calculate the gonadosomatic index.
Data source location	Ob River basin (66°46′3″ - 57°21′01.7″N, 63°33′27.2″ - 72°00′27.9″E), Tyumen Region, Russia
Data accessibility	Raw data are available in the dataset “Length, weight, sex, gonad weight and maturity stage of Rutilus rutilus and Perca fluviatilis from the Ob River basin, Western Siberia” [2]. Repository name: Mendeley Data. Data identification number: DOI: 10.17632/3sp3sj4z8m.1 Direct URL to data: https://data.mendeley.com/datasets/3sp3sj4z8m/1 Analyzed data are available with the article (Supplementary data).

## Value of the Data

• The data contribute to the knowledge of given fish species biology and provide new cases for meta-analysis of length-weight, length-length, or weight-weight data.
• Specialists involved in fish stock assessment can benefit from these data.
• Length-weight relationships are helpful in gaining data on fish growth and biomass.
• The data can be used to calculate the relative weight *W*_rm_ [3] or other length-/ weight-related indices for comparing the condition of fish across populations.

## 1 Data Description

**Fig. 1:**
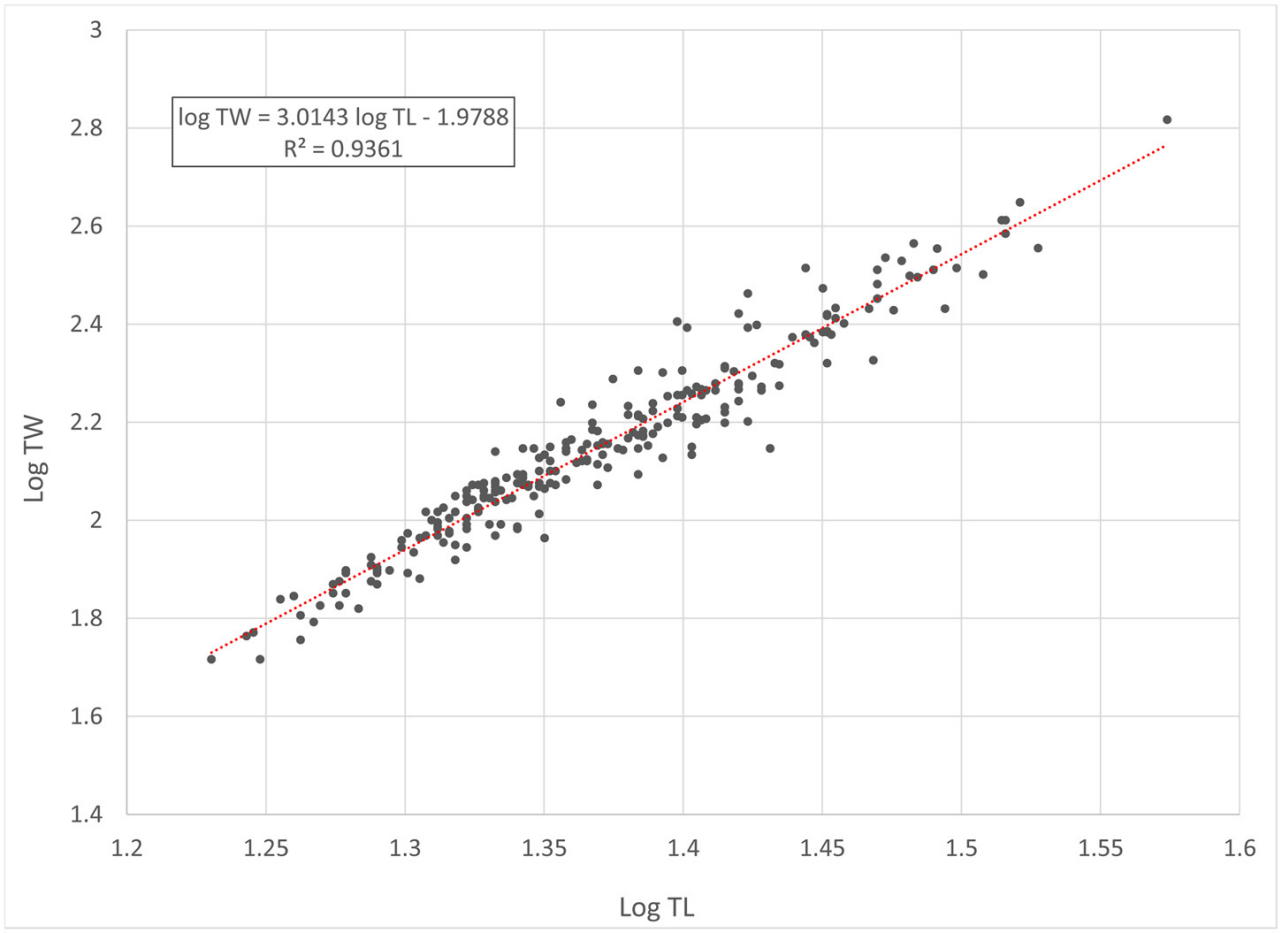
Linear regression analysis of log-transformed TL and TW of roach *Rutilus rutilus*.

**Fig. 2:**
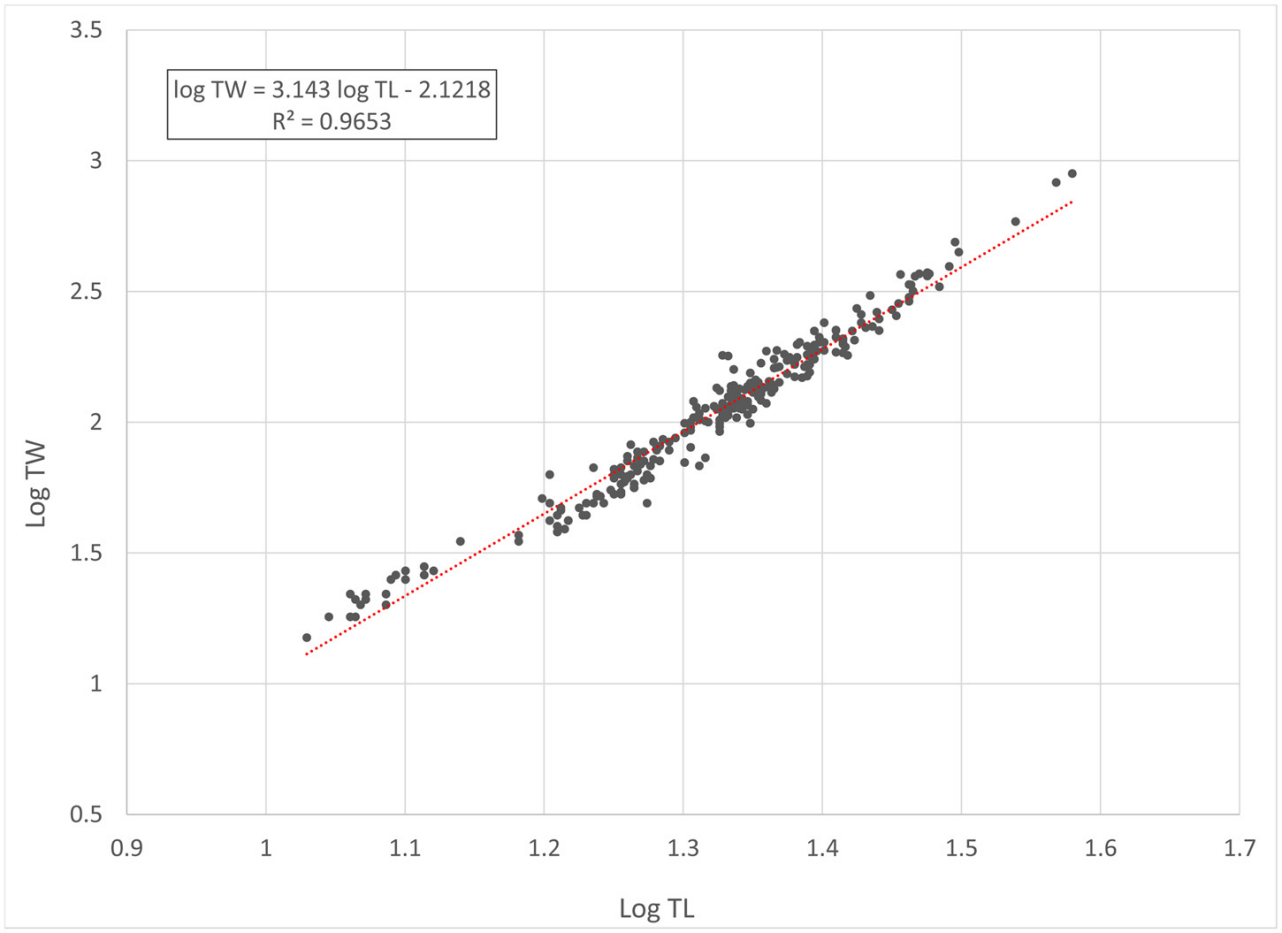
Linear regression analysis of log-transformed TL and TW of European perch *Perca fluviatilis*.

**Fig. 3:**
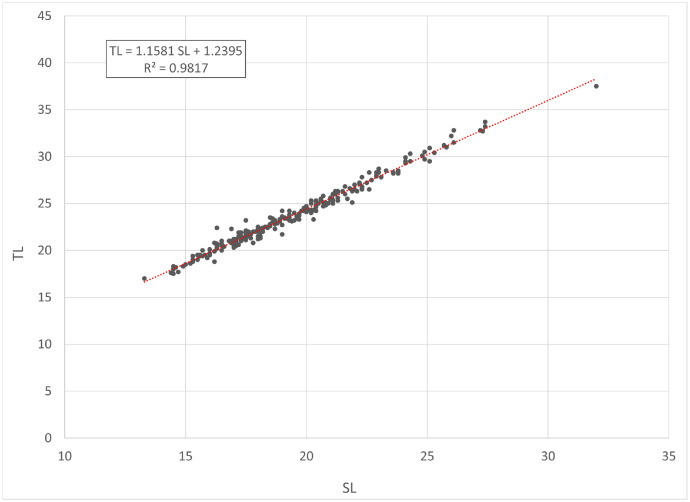
Linear regression analysis of SL and TL of roach *Rutilus rutilus*.

**Fig. 4:**
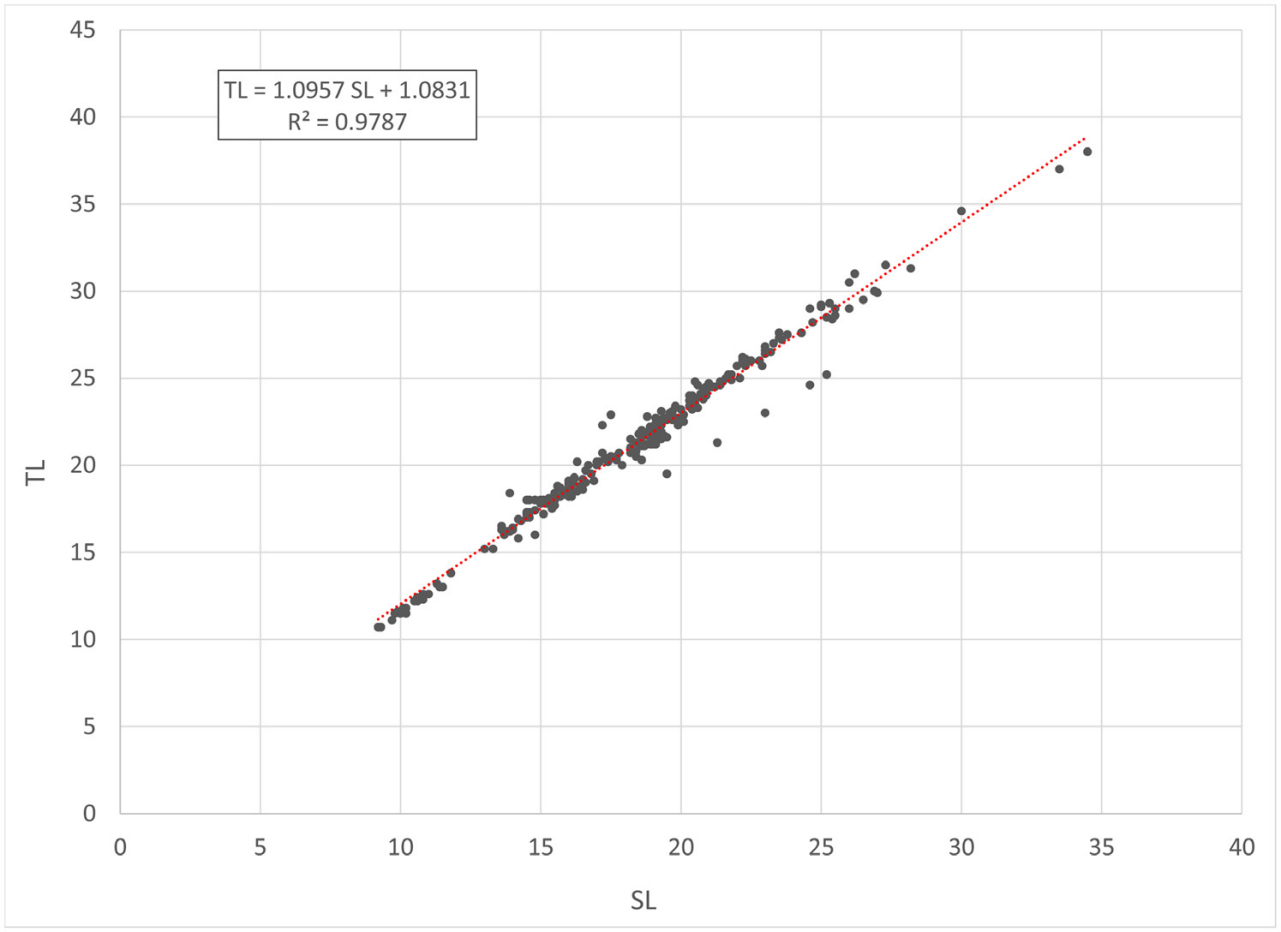
Linear regression analysis of SL and TL of European perch *Perca fluviatilis*.

Fig. 1 and Fig. 2 present linear regression models for the log-transformed raw data on the total length (TL) and total weight (TW) of roach *Rutilus rutilus* and perch *Perca fluviatilis*. Descriptive statistics and estimated parameters of length-weight relationships are given in Table 1. Fig. 3 and Fig. 4 show length-length relationships derived from the raw TL and standard length (SL) data. Sampling dates and locations, raw data related to Fig. 1, Fig. 2, Fig. 3, Fig. 4, sex and gonad maturation stage, mean condition factor (CF_mean_), and gonadosomatic index (GSI) values are reported in spreadsheets and given in Supplementary Data 1.

**Table 1:** Descriptive data and length-weight relationship parameters for roach *Rutilus rutilus* and perch *Perca fluviatilis*.

		TL, cm		TW, g						LWR	
Species	*N*	Min	Max	Min	Max	*a*	95% CL of *a*	*b*	95% CL of *b*	TW = *a* TL*^b^*	*R*^2^
*Rutilus rutilus*	250	17.0	37.5	52	656	0.0105	0.0077-0.0143	3.0143	2.9163-3.1123	TW = 0.0105 TL^3.0143^	0.936
*Perca fluviatilis*	274	10.7	38.0	15	892	0.0076	0.0061-0.0094	3.1430	3.0722-3.2138	TW = 0.0076 TL^3.1430^	0.965

Note: *N* – number of specimens used for the linear regression analysis; TL – total length; TW – total weight; CL – confidence limit; LWR – length-weight relationship; *R*^2^ – coefficient of determination of the linear regression.

## 2 Experimental Design, Materials and Methods

Fish specimens were collected in 2011 and 2012 in the Ob River basin using gill-nets with 22 and 28 mm mesh sizes. Each individual, one after the other, was immobilized by a stunning blow to the head, quickly measured in TL and SL to the nearest 0.1 cm using a measuring board. Then, TW was determined, and a fish was subjected to cervical transection. Next, each specimen was dissected, and the sex and gonad maturity stage were determined visually. Gonads were excised and weighed (GW) to the nearest 0.1 g. After that, abdominal and pericardia cavities were cleared, and the eviscerated weight (EW) of a fish was measured. TW and EW were determined with an accuracy of 1 g using an Adam HCB 1002 digital balance.

In total, raw data on 250 specimens of *Rutilus rutilus* and 274 specimens of *Perca fluviatilis* were collected. Length-weight relationships were calculated by the linear regression of log-transformed total weight on total length [3]. Microsoft Office Excel was used for processing the data. Log_10_ values of TL and TW were used to draw a scatter plot for each species. A trend line was added to each scatter plot to obtain a linear-regression equation (log TW = *b* log SL + log *a*). Log *a* values were back-transformed to find a length-weight relationship (TW = *a* SL*^b^*). Further, TL and SL were used to find a length-length relationship by linear regression. CF_mean_ values were calculated according to the formula: CF_mean_ = 100 *a* SL*^b^*^-3^ [3]. GSI values were obtained using the formula: GSI = 100 GW TW^−1^.

## Ethics Statement

Data collection complied with the ARRIVE guidelines and was carried out in accordance with the U.K. Animals (Scientific Procedures) Act, 1986 and associated guidelines, EU Directive 2010/63/EU for animal experiments.

## CRediT Author Statement

**Leonid Shuman:** Investigation, Data curation, Writing – review & editing; **Aleksandr Selyukov:** Investigation, Data curation, Writing – review & editing; **Innokenty Nekrasov:** Investigation, Data curation; **Andrey Elifanov:** Investigation, Data curation; **Victoria Yurchenko:** Formal Analysis, Writing – original draft, Writing – review & editing.

## Declaration of Competing Interest

The authors declare that they have no known competing financial interests or personal relationships which have or could be perceived to have influenced the work reported in this article.

## Data Availability

Length, weight, sex, gonad weight and maturity stage of Rutilus rutilus and Perca fluviatilis from the Ob River basin, Western Siberia (Original data) (Mendeley Data).

## Appendix Supplementary materials

Image, application 1

## Footnotes

Supplementary material associated with this article can be found in the online version at doi:10.1016/j.dib.2022.108067.
